# Structural adjustment programmes adversely affect vulnerable populations: a systematic-narrative review of their effect on child and maternal health

**DOI:** 10.1186/s40985-017-0059-2

**Published:** 2017-07-10

**Authors:** Michael Thomson, Alexander Kentikelenis, Thomas Stubbs

**Affiliations:** 1 School of Social Sciences, University of Waikato, Hamilton, New Zealand; 2 Trinity College, University of Oxford, Oxford, UK; 3 Department of Sociology, University of Amsterdam, Amsterdam, The Netherlands; 4 Centre for Business Research, University of Cambridge, Cambridge, UK

**Keywords:** Structural adjustment, Child health, Maternal health, International financial institutions, International Monetary Fund, World Bank, African Development Bank

## Abstract

Structural adjustment programmes of international financial institutions have typically set the fiscal parameters within which health policies operate in developing countries. Yet, we currently lack a systematic understanding of the ways in which these programmes impact upon child and maternal health. The present article systematically reviews observational and quasi-experimental articles published from 2000 onward in electronic databases (PubMed/Medline, Web of Science, Cochrane Library and Google Scholar) and grey literature from websites of key organisations (IMF, World Bank and African Development Bank). Studies were considered eligible if they empirically assessed the aggregate effect of structural adjustment programmes on child or maternal health in developing countries. Of 1961 items yielded through database searches, reference lists and organisations’ websites, 13 met the inclusion criteria. Our review finds that structural adjustment programmes have a detrimental impact on child and maternal health. In particular, these programmes undermine access to quality and affordable healthcare and adversely impact upon social determinants of health, such as income and food availability. The evidence suggests that a fundamental rethinking is required by international financial institutions if developing countries are to achieve the Sustainable Development Goals on child and maternal health.

**Electronic supplementary material:**

The online version of this article (doi:10.1186/s40985-017-0059-2) contains supplementary material, which is available to authorized users.

## Background

In the past four decades, structural adjustment programmes administered by international financial institutions (IFIs), such as the International Monetary Fund (IMF), World Bank, and regional development banks, have typically set the fiscal parameters within which health policies operate in developing countries. These programmes gained notoriety among public health advocates following the publication of UNICEF’s seminal ‘adjustment with a human face’ [1], which found adverse child and maternal health outcomes attributable to the means by which economic adjustment had been implemented. Several studies have since found adverse health effects associated with structural adjustment [2–12].

Structural adjustment loans are provided to countries in dire fiscal or macroeconomic straits. In return, recipient countries are required to reform various macroeconomic and fiscal policies according to a neoliberal rubric, typically cohering around economic stabilisation, trade and financial liberalisation, deregulation, and privatisation [13]. Collectively, these ‘conditionalities’ are purposed with ensuring states are capable of servicing debt, as well as setting the economic climate for growth. However, critics argue such adjustment comes at a high social cost, while the recidivist nature of program participation also suggests that gains to macroeconomic stability are underwhelming [14, 15].

IFIs contend that their programmes promote health by increasing revenues available for health spending via economic growth [16–18], safeguarding government health spending from fiscal consolidation [18, 19], and catalysing health aid through signals to foreign aid organisations and investors of sound fiscal management [16]. Conversely, critics argue that rigid fiscal targets stipulated under structural adjustment loans often take precedence over social spending, and that aid funds are siphoned from health and social sectors to repay debt or increase reserves [22–27]. The notion that IMF fiscal consolidation is conducive to growth is likewise contested [28, 29], with implications on revenues available for health spending.

These unresolved debates remain relevant as the global community mobilises to achieve the Sustainable Development Goals (SDGs), which target vast reductions in maternal, under-5, and neonatal mortality rates by 2030. Specifically, SDG 3.1 aims to reduce the global maternal mortality ratio to less than 70 per 100,000 live births, while SDG 3.2 aims for both a neonatal mortality of less than 12 per 1000 live births and an under-5 mortality rate of less than 25 per 1000 live births [30]. Despite significant advances made to meet the preceding Millennium Development Goals (MDGs), efforts fell short of the targeted two-thirds reduction of under-5 mortality and three-quarters reduction of maternal mortality between 1990 and 2015. Estimates in 2015 placed the worldwide infant mortality rate at 32 per 1000 live births, under-5 mortality at 43 per 1000 live births, and maternal mortality at 216 per 100,000 live births [30, 31]. Developing regions accounted for 98.7% of under-5 deaths in 2015, with Sub-Saharan Africa alone bearing 49.6% of the global total [30]. While these indicators have improved rapidly since the 1960s, the pace of improvement decelerated in the 1990s, even as other parts of the world experienced significant gains [32]. Could the slow-down be attributed, at least in part, to the policy prescriptions attached to structural adjustment programmes?

If the claims of critics are true, then a significant rethinking of structural adjustment programmes is required if the SDGs are to be achieved. Academic literature, however, does not adequately explain how structural adjustment impacts maternal and child health. Prior reviews have focused on much more broadly defined populations [33], or are non-systematic [10]. To our knowledge, this article is the first to narrow its scope to child and maternal health. These populations are often vulnerable to macroeconomic policies in ways distinct from the broader population [4, 7]. Further, there is a tendency in existing research to treat structural adjustment as a homogeneous intervention, despite the relative heterogeneity of policy packages entailed [22]. Interpreting the relationship between structural adjustment and health can be greatly enhanced through the identification of specific mechanisms that influence health outcomes [34].

We thus conduct a systematic-narrative review to assess empirical evidence of effects to child and maternal health resulting from structural adjustment administered by the IMF, World Bank, and African Development Bank (AfDB). To better understand the disaggregated mechanisms producing net health effects, we supplement the systematic review with a synthesis of conceptual understandings of the subsidiary pathways linking structural adjustment to child and maternal health outcomes.

We begin by outlining the history of structural adjustment. Subsequently, we describe the search strategy employed to survey the literature. In our findings, we present evidence of the net effect of structural adjustment on child and maternal health outcomes. We then discuss the results by exploring the specific pathways via which this effect takes place. We conclude by summarising key findings, assessing study limitations, examining directions for future research, and highlighting implications for SDG attainment.

### A brief history of structural adjustment

Foremost among IFIs are the IMF and World Bank [35], which act as lenders to countries requiring financial assistance. Through their ‘conditionalities’—or policy reforms required to receive loans—IFIs maintain a powerful bargaining position from which to influence domestic policy.

The IMF and World Bank were established in the Bretton Woods conference of 1944, with respective mandates to maintain international financial stability and finance development projects. While early operations were confined to specific macroeconomic targets such as expenditure ceilings, by 1974 and 1980, respectively, the IMF and World Bank integrated reforms intended to fundamentally restructure recipient economies [36, 37]. These organisations came to embody a ‘Washington Consensus’ of ‘neoliberal’—or market-led—growth strategies, to be promoted globally via both direct stipulations in loan agreements and advisory influence more generally [38]. Thus, over the course of the 1980s, the Bretton Woods twins transitioned from fiscal crisis and infrastructural creditors to arbiters of the broad-scale direction of global economic and social policy.

Early structural adjustment programmes promulgated across low- and middle-income nations during the debt crises of the 1980s. Reform packages cohered around four central principles of neoliberalism: economic stabilisation, liberalisation, deregulation, and privatisation [13]. *Stabilisation* refers to policies which seek to limit fluctuations in exchange rates, inflation, and balance-of-payments. *Liberalisation* encompasses measures designed to facilitate the free flow of trade and capital, such as the removal of tariffs. *Deregulation* involves the removal of governmental ‘red-tape’ vis-à-vis business practises, such as stipulations in employment relations law. Finally, *privatisation* describes the transferal of enterprise from state to private ownership, thereby fostering competition and market efficiencies. In response to extensive criticism of this model of development during the 1990s [39], the IMF and the World Bank purport to have shifted their orientation by incorporating ‘pro-poor’ measures to their programme design [40, 41]. Yet, recent studies find this ostensible shift to have changed little in practice [22]. Contrary to the rhetoric, conditionalities continue to advance a neoliberal conception of economic development [22, 32].

Regional development banks have offered little alternative to the precedent set by the Bretton Woods twins. In Sub-Saharan Africa—the region accounting for the greatest proportion of structural adjustment programmes [8]—the AfDB fulfils a similar function to its global counterparts. The AfDB was founded in 1964 by 35 African nations intent on solving the continent’s problems internally [2, 3, 9]. However, the oil price hikes of the 1970s eroded its capital sharply and produced massive debt among member nations, compelling the AfDB to gradually favour structural adjustment lending over project lending to ensure debts would be repaid [9]. Despite its early intention to maintain an African character, in practice its loans bear little point of distinction from those administered by the IMF and World Bank. Indeed, these institutions co-finance some 90% of AfDB loans, and the organisation is heavily influenced by shared expertise and funding pressures to follow the lead of the Bretton Woods twins [42].

## Methods

We systematically review four electronic databases, with additional documents from the websites of the IMF, World Bank, and AfDB, to synthesise empirical evidence and hypotheses on the relationship between IFIs and child and maternal health in the developing world. The review was conducted in accordance with PRISMA guidelines [43, 44]. A full PRISMA checklist is provided in Additional file 1.

### Selection criteria

We consider empirical aggregate-effect studies on structural adjustment programmes conducted by three international organisations: the IMF, World Bank, and AfDB. The two former were selected by virtue of administering the greatest number of adjustment programmes globally [37, 45], while the AfDB’s inclusion reflects its extensive involvement in implementing programmes in Sub-Saharan Africa—a region with both the largest number of such programmes and highest rate of under-5 and maternal mortality in the world [8, 30, 31]. We define children as individuals below age 18, and maternity as beginning with pregnancy and ending 6 weeks postpartum, as recommended by the World Health Organization [46]. Emerging market and developing nations were classified according to the IMF’s World Economic Outlook Report, October 2016 [47].

### Search strategy

Academic articles were sourced from four electronic databases (PubMed/Medline, Web of Science, Cochrane Library, and Google Scholar), as well as by scanning reference lists. Supplementary grey literature was located by searching the websites of the IMF, World Bank, and AfDB, as well as via Google Scholar. Table 3 in Appendix demonstrates the full search strategy used for PubMed/Medline. Strategies for the other databases are identical in substance, with minor adjustments to suit the idiosyncrasies of each search engine.

Our pilot database search limited results to English language texts published from January 2008 onward with human subjects. The final search, conducted in March 2017, amended the inclusion date to 2000 to increase sensitivity. The search strategy combined three term categories (interventions, outcomes, and setting) phrased in the National Library of Medicine’s hierarchically organized standardised Medical Subject Headings (MeSH) indexing terms, and in plain text for sensitivity. Intervention key terms included the names of IFIs and policy levers associated with structural adjustment. Outcome key terms included various indices of mortality, pregnancy complications, school absenteeism, diseases and other health conditions, and broad measures of health and wellbeing for fetal, infant, child, and maternal populations. Setting key terms covered clustered geographical areas coded in MeSH terms. Where MeSH terms were not available, additional terms were added to maintain sensitivity. For instance, in lieu of the proliferating MeSH category “pregnancy complications”, lower-tier terms such as “stillbirth” were added manually to the search.

Search strategies on the websites of IFIs were adapted to suit the less sophisticated search functionality. Intervention keywords included “structural adjustment” and “conditionality”, while outcome variables included “infant mortality”, “child mortality”, “maternal mortality”, and “health”. Articles were screened in three phases. First, texts were downloaded to Endnote X7 if their title and abstract appeared relevant to the research question. Second, article abstracts were screened against the selection criteria outlined above, barring restrictions on study design. Third, full-text screening distinguished empirical studies for systematic review from conceptual and review articles to be retained for subsequent discussion, and further eliminated texts with misleading relevancy to the selection criteria, or only a secondary focus on the research question of this review.

### Search results

**Fig. 1:**
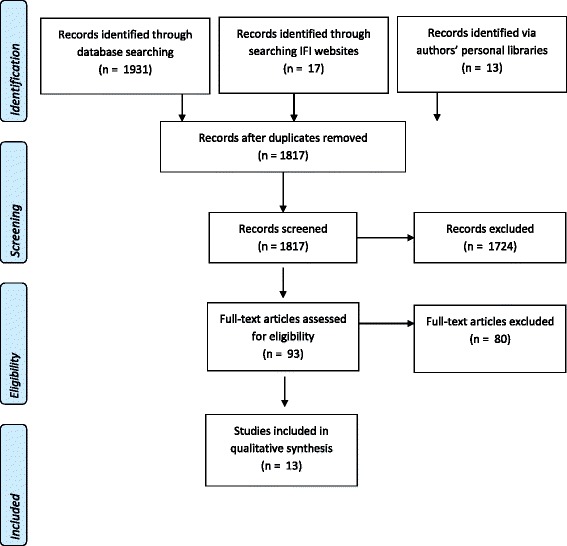
PRISMA flowchart for academic literature search

Figure 1 displays the results of the systematic review. A total of 1931 records were downloaded to EndNote X7 following the initial database search. A further 13 texts were identified through scanning reference lists, and searching IFI websites yielded another 17 entries. 1817 abstracts were screened for relevance after the exclusion of 144 duplicates. Ninety-three texts were obtained for full-text screening, and 13 were identified as meeting the inclusion criteria.

A standardised format for data extraction was established a priori, collating the study’s aim, hypothesised pathways, study period, research design, main findings, and limitations. An important methodological consideration in this review was the extent to which these studies distinguish programme effects from selection effects. IFI programmes are not random events, as typically only countries whose economies experience severe economic and financial difficulties participate [3, 48]. Studies failing to adequately control for initial conditions faced by countries—including unobserved factors like political will of the government—will thus confound the effect of adjustment with the underlying factors prompting participation in the first place. Scholars typically employ four econometric strategies to overcome selection bias: matching methods, instrumental variables approaches, system GMM estimation, and Heckman selection models [48].

#### Findings

In this section, we review empirical evidence on the effect of structural adjustment programmes on child and maternal health respectively.

### The effect of structural adjustment on child health

Table 1 summarises studies examining the effects of structural adjustment on child health outcomes. Eight of the ten studies found a detrimental relationship between structural adjustment and child health outcomes, while one found no association and one established a beneficial effect.

**Table 1:** Characteristics of studies on the aggregate effect of structural adjustment programmes on child health

	Outcome measure	IFI	Study period	Sampling	Study setting	Addresses selection bias	Analysis	Effect of structural adjustment
Coburn, Restivo and Shandra, 2015	Infant mortality	AfDB	1990–2006	5-yearly	31 African countries	Yes	Fixed-effects OLS regression	Detrimental
Shandra, Shandra and London, 2012	Infant mortality	IMF	1990–2005	5-yearly	32 African countries	Yes	Fixed-effects OLS regression	Detrimental
Hajro and Joyce, 2009	Infant mortality	IMF	1985–2000	5-yearly	82 developing countries	Yes	Fixed-effects OLS regression	Beneficial for non-concessionary loans in interaction with economic growth and no association for concessionary loans
Shandra, Nobles, London and Williamson, 2004	Infant mortality	IMF	1980–1997	Two-point	59 developing countries	No	Lagged dependent panel regression	Detrimental in interaction with democracy
Noorbakhsh and Noorbakhsh, 2006	Infant mortality	World Bank	1975–1998	5-yearly	33 African countries	No	OLS regression	No association
Oliver, 2006	Infant mortality and under-5 mortality	IMF and World Bank	1980–2000	N/A	Argentina and Uruguay	No	Comparative case study	Detrimental
Pandolfelli and Shandra, 2013	Child mortality	AfDB	1990–2005	5-yearly	35 African countries	Yes	Random-effects generalised least-squares regression	Detrimental
Shandra, Shandra and London, 2011	Child mortality	World Bank	1990–2005	5-yearly	31 African countries	No	Fixed-effects OLS regression	Detrimental
Pongou, Salomon and Ezzati, 2006	Child malnutrition and maternal health-seeking behaviours	IMF and World Bank	1991–1998	Pooled two-point	1587 and 1923 children in Cameroon	No	OLS regression	Detrimental
Daoud, Nosrati, Reinsberg, Kentikelenis, Stubbs and King, 2017	Child malnourishment	IMF	c2000	Cross-sectional stratified	67 developing countries, with 1, 941, 734 children	Yes	Multi-level logistic regression	Detrimental in interaction with parental education

We begin with studies yielding detrimental effects. Two cross-country quantitative studies of Sub-Saharan African nations from the period 1990 to 2006 find a positive relationship between infant mortality and the presence of an AfDB and IMF structural adjustment loan, respectively [2, 49]. Both studies explicitly control for a standard battery of initial conditions, including a series of domestic health, political, and economic factors, as well as two-way fixed effects. To account for non-random selection into the programmes, the former uses a two-step Heckman selection approach and the latter deploys an instrumental variable approach, both of which are well-established procedures in the literature [48]. A third study examines the impact of AfDB structural adjustment loans on child mortality for Sub-Saharan African nations for the same period using generalised least square random effects regression models within a two-step Heckman procedure, again finding a positive relationship [9]. The study attributes an additional 85.62 under-5 deaths per 1000 to structural adjustment programmes administered by the AfDB. Fourth, a recent study deploys multi-level modelling techniques to investigate the effect of IMF structural adjustment loans on child malnutrition in 67 countries for almost two million children for circa year 2000 [50]. Controlling for non-random selection using a two-step Heckman approach, the study finds no direct effects of IMF programmes on child malnourishment; however, when adding a set of interaction terms, it finds the presence of an IMF programme decreases the protective effect of parents’ education on child malnourishment by at least 17%. The study claims this is due to IMF reforms that make it harder for parents to reap the benefits of their education, such as wage contraction and welfare retrenchment.

A further four studies assess child health outcomes without addressing non-random selection into programmes. One tests the impact of World Bank structural adjustment on child mortality across sub-Saharan African nation for 1990 to 2005 using two-way fixed effects regression models, finding a positive association between the two [12]. Another exploits a quasi-experimental design using pooled cross-sectional data from two Demographic and Health Surveys conducted in 1991 and 1998 to measure changes in childhood malnutrition in response to World Bank and IMF structural adjustment in Cameroon [11]. The authors attribute greater levels of malnutrition in children born between 1995 and 1998 than those born between 1988 and 1991 to government health expenditure cuts experienced during structural adjustment programmes between 1992 and 1994. In addition, a comparative case study compares the effects of World Bank and IMF structural adjustment on health in Argentina and Uruguay [51]. It finds that structural adjustment was implemented with greater severity and speed in Argentina than in Uruguay, and that the more gradual and modest reforms in Uruguay were associated with better health outcomes: Uruguay’s infant and under-5 mortality rates declined at twice that of Argentina’s throughout the 1980s. However, the study is limited by its inability to isolate the contribution of structural adjustment from confounding factors, such as the extent of the underlying economic crisis and the political will of the government. Finally, a study examines the effects of IMF structural adjustment on infant mortality based on a lagged dependent variable panel regression on a sample of 59 developing countries in 1997 [52]. It finds no effect for the IMF variable in isolation. However, the interaction between the IMF variable and political democracy yielded a detrimental effect on infant mortality, which was greater at lower levels of democracy than at higher levels.

Only one study finds no association between structural adjustment and child health outcomes [53]. It examines the relationship between compliance with World Bank conditions—including macroeconomic stabilisation polices, public sector management, and private sector development—and infant mortality across Sub-Saharan African countries in 5-year periods from 1980 to 2001, but did not account for non-random country selection into programmes.

Another study finds a beneficial relationship between adjustment and child health [5]. Investigating the effect of the IMF’s non-concessionary and concessionary programmes—that is, low interest loan facilities to low-income countries—for 82 developing countries during the period 1985 to 2000, it finds neither have a direct effect on infant mortality. The study then interacts the IMF variables with growth, finding growth that occurs under concessional loans results in an additional decline in infant mortality of 0.4 per 1000 infants. However, the study does not correct for non-random country selection into structural adjustment: it erroneously claims that its two-way fixed effects approach adequately addresses these methodological concerns.

### The effect of structural adjustment on maternal health

Table 2 summarises studies investigating the effect of structural adjustment on maternal health outcomes. The three studies—all having one co-author in common—show that structural adjustment has an adverse impact on maternal mortality.

**Table 2:** Characteristics of studies on the aggregate effect of structural adjustment programmes on maternal health

	Outcome measure	IFI	Study period	Sampling	Study setting	Addresses selection bias	Analysis	Effect of structural adjustment
Coburn, Restivo and Shandra, 2015	Maternal mortality	AfDB	1990–2005	5-yearly	31 African countries	Yes	Random-effects generalised least-squares regression	Detrimental
Shandra, Shandra and London, 2015	Maternal mortality	IMF	1990–2000	5-yearly	65 developing countries	No	Lagged dependent panel regression	Detrimental
Pandolfelli, Shandra and Tyagi, 2014	Maternal mortality	IMF	1990–2005	5-yearly	37 African countries	Yes	Random effects generalised least-squares regression	Detrimental

Two studies deploy a cross-country regression design with Sub-Saharan African samples for the period 1990 to 2005 [3, 8]. Deploying a two-step Heckman procedure to account for non-random selection, both studies find detrimental changes to maternal mortality associated with IMF and AfDB structural adjustment loans, respectively. The former reports that an additional 360 maternal deaths per 100,000 live births are attributable to IMF structural adjustment; while the latter shows that approximately 231 additional maternal deaths per 100,000 live births are attributable to AfDB structural adjustment. A final study analyses a sample of 65 developing countries for 2005 using lagged dependent variable panel regression, finding a positive relationship between structural adjustment and maternal mortality [54]. However, the study design does not account for selection bias.

## Discussion

The empirical studies identified in our systematic review are virtually unanimous in finding a detrimental association between structural adjustment and child and maternal health outcomes. However, these studies treat structural adjustment as a ‘black box’—assessing its aggregate effect on child and maternal health outcomes rather than delineating pathways. Identifying plausible mechanisms is also important insofar as there are some aspects of structural adjustment that are beneficial to health outcomes, even while the net effect is detrimental. Applying Kentikelenis’ framework for assessing the potential health effects of structural adjustment programmes, we organise the mechanisms linking IFI programmes with child and maternal health outcomes into (a) those mediated via direct effects on health systems, (b) those mediated via indirect effects on health systems, and (c) those related to the social determinants of health [34]. Pathways discussed in this section include those hypothesised in the empirical studies reviewed above, as well as additional empirical, conceptual, and review articles identified through the literature search process.

### Changes to child and maternal health via direct effects on health systems

Policies adopted in adherence with structural adjustment programmes frequently bear consequence to the functioning of health systems, with implications for child and maternal health outcomes. First, structural adjustment is hypothesised to affect *government health expenditure*, which in turn alters the quality and quantity of services provided to children and mothers [2, 3, 9, 12, 24, 49, 54, 55]. Governments may be under explicit or implicit pressure to cut social spending in order to meet fiscal targets, thereby reducing the fiscal space in which healthcare systems can operate [8, 55–57]. Consequently, countries experience medical supply shortages [6], loss of human capital [58], and replacement of defunded maternal health services with ineffective traditional birth attendant programs [10]. One study found that reduced government funding weakened health services, such that responses to HIV/AIDS in Sub-Saharan Africa were significantly impaired [59]. Empirical studies assessing the effect of health expenditures or government spending more broadly find a significant and detrimental relationship with infant mortality [58, 60, 61], under-5 mortality [58], and other health outcomes [62]. IFI-affiliated authors contest the notion that structural adjustment programmes reduce health spending [63] or claim they are associated with increased spending [19, 20, 64, 65]. Conversely, independent scholars tend to present a conditional account in which spending increases only in Sub-Saharan African low-income countries and autocracies, while decreasing in other low-income settings [24].

Structural adjustment can similarly affect the *healthcare workforce*, thereby altering the quality and quantity of healthcare staff available to treat child and maternal health conditions [7, 66]. Adjustment programmes may include conditions that specify ceilings on the public sector wage bill, which can force government cuts to wages and personnel in the healthcare sector [66]. Reduced wages and job security often creates incentives for health workers to move elsewhere, producing ‘brain drain’ [7]. In 2007, the IMF changed their wage bill ceiling policy in recognition of its adverse effects [19, 67, 68] and have argued this issue no longer stands [64, 69]. Nevertheless, wage bill ceilings remain a persistent, if subtle, feature of recent programmes [22].

Structural adjustment programmes frequently introduce *cost-sharing* or *user fees* to enhance the fiscal sustainability of healthcare services [4, 70]. While fee introduction can increase the range of services available to middle classes and wealthy elites, they can greatly reduce access to even the most rudimentary health services for the poor [4, 6, 56, 71]. A World Bank directive to introduce a US$0.33 charge for outpatient health centre visits saw a 52% reduction in visits, followed by a 41% recovery when user fees were suspended [59]. Furthermore, user fees are associated with greater incidence of stunted growth in children [57], dramatic reductions in women’s use of STI clinics [4], and barriers to access for antimalarial medication and antibiotics [70]. A design simulation model of 20 African countries employing user fees for health concluded that abolition of fees could prevent an estimated 233,000 under-5 deaths annually or 6.3% of such deaths in these settings [70]. As per wage bill ceilings, user fees are no longer endorsed by IFIs [71].

IFIs commonly prescribe changes to the *public-private mix* in the health sector. Increasing private provision of health services is hypothesised to broaden access to services for the middle and upper classes, but raises financial barriers for poor women and children as providers shift to a profit-driven business model [8, 9, 54].

IFIs also endorse state retrenchment in the provision of healthcare and other services to promote a *greater role for non-governmental organisations* (NGOs) [10, 54]. An empirical study on the link between the increasing role of NGOs in health provision and maternal mortality rates found support for what the authors term the “political opportunity structure hypothesis”, whereby NGO provision of healthcare produces greater reductions in maternal mortality as nations become more democratic. According to this account, popular mandates increase the leverage that civil society organisations wield in relation to government decision-making, thereby increasing their capacity to influence health spending [54]. While this may suggest NGOs are an adequate substitute for public healthcare in democratic settings, the study was severely limited by data availability.

Similarly, adjustment programmes commonly promote *decentralisation of health systems* in favour of increased local autonomy [34]. Decentralised systems allow services to address region-specific demands, but may produce a more fractious and unequal implementation of services—including those for child and maternal health—nationally. Furthermore, lack of co-ordination in decentralised systems can hinder efforts to combat major disease outbreaks [23].

Finally, in recent years IFIs have made an increasing effort to include *priority spending floors,* which protect health spending from fiscal consolidation [21, 65, 72, 73]. IFI-affiliated authors claim that these floors have increased access to, and supply of, health services—including those for children and mothers—by ring-fencing health spending [72]. In support of this appraisal, archival evidence on IMF programmes in West African nations shows that, in select instances, priority spending floors contributed to increases in budgetary allocations for health, as was the case for Gambia in 2012 and Benin in the late 1990s [27, 55]. As noted above, fund programs are also associated with higher health expenditures in Sub-Saharan African low-income countries, which historically spent less than any other region [24]. However, despite some successes, the evidence shows social spending targets are upheld less than half the time, while fiscal targets are rarely breached [22, 23].

### Changes to child and maternal health via indirect effects on health systems

The effects of structural adjustment policies on health systems are often indirect. One mechanism by which health systems are indirectly affected is via *currency devaluation*. Devalued currencies promote export competitiveness, but increase the real cost of imports, including pharmaceutical goods and health equipment [4, 6, 8, 49], which may plausibly have negative implications for child and maternal health outcomes; however, we identified no empirical studies verifying the link.

Structural adjustment programmes also promote *trade and capital account liberalisation* measures, such as the removal of tariffs and capital controls, to encourage growth and foreign direct investment. While tax revenues can increase in the long run if these measures stimulate growth, scholars raise concerns about both the short-run loss of tariff revenue available for healthcare and the long-term repatriation of profits by multinationals receiving tax holidays [6, 8, 9, 12, 49, 52, 53]. One study reports that the mass migration of smallholder farming families to urban areas caused by aggressive trade liberalisation policies was a major contributor to the HIV epidemic in Sub-Saharan Africa [4]. Despite claims by critics to the contrary, IFIs maintain they are not ideologically predisposed to trade liberalisation [74].

*Privatisation* outside the health sector can have indirect influences on health systems as well. The sale of state-owned enterprises may produce a windfall in the short-term, but the cumulative loss of profits from such businesses reduces government revenues in the mid-term. Accordingly, fewer resources are available to finance healthcare subsidies and services for children and mothers [4, 49, 59]. Privatisation may also result in public sector job loss that is not necessarily substituted by the establishment of new positions in the private sector. For example, more than 150,000 workers were displaced when Ghana privatised 42 of its largest state enterprises between 1984 and 1991. Such unemployment disproportionately affects women, who are likely to be lower skilled and made redundant, which in turn increases commercial sex uptake, and—due to greater risks of contracting STIs—can lead to complications during child birth [4].

In addition, countries receiving structural adjustment loans must devote government revenue to facilitate *debt servicing*. Unless protected or substituted via external sources, resources devoted to debt servicing may impinge upon health sector budgets, thereby reducing spending dedicated to improving child and maternal health outcomes [2, 3, 8, 9, 12, 49, 53]. While this association seems plausible, we identified no empirical studies investigating the connection.

Finally, structural adjustment programmes can catalyse aid inflows by signalling to donors that a country possesses sound governance and fiscal management [34]. These increased inflows may help to offset negative effects on child and maternal health outcomes by channelling resources back into healthcare provision. Indeed, a doubling of health aid is associated with a 2% reduction in infant mortality rate [75]. However, a recent study examining the types of aid catalysed by IFI programmes found no significant effect on health aid inflows [76].

### Changes to child and maternal health via effects on social determinants

Structural adjustment policies may influence child and maternal health in ways which bypass health systems, and instead act upon the social determinants of health [77]. One example is the *increased reliance on unsanitary water* accompanying increasing privatisation and deregulation. Water and sanitation facilities under private ownership may introduce unaffordable fees for water access, leading the poor to rely on water from degraded sources. Pathogens in such waterways can lead to diarrhoea infections, which disproportionately affect children, while improved water sources and sanitation both improve child mortality by removing exposure to such pathogens [12].

Trade liberalisation and currency devaluation can lead to a *rising real price of food*, which in turn reduces maternal and child nutritional intake [56]. A World Bank study into the link between commercialisation of agriculture and child malnutrition in Malawi found that children who came from households dependent on cash crop production were more vulnerable to stunting in response to food price shocks than those from less reliant households [78]. This implies that dependence on cash crop production for subsistence magnifies vulnerability to global market conditions, to the detriment of child nutrition. Liberalisation has also been linked to a ‘nutrition transition’ owing to the penetration of multinational supermarkets and fast food brands, leading to the double burden of both malnutrition *and* obesity in the same settings [79].

IFI fiscal consolidation policy justifies *short-run economic contraction* on the grounds that resolving balance-of-payment issues and transitioning to a model of export-oriented, private sector-led growth will maximise economic growth in the long run. However, increased short-run unemployment may reduce income available to pay for healthcare even as privatisation and user fees increase the cost of services [34]. Moreover, IFIs may miscalculate the duration and depth of fiscal contraction. The IMF’s own Independent Evaluation Office noted a “tendency to adopt fiscal targets based on overoptimistic assumptions about the pace of economic recovery” (p. vii), thus multiplying the negative impact of economic contraction [69]. Further, the ubiquity of export-led growth strategies worldwide as per the Washington Consensus may constitute a fallacy of composition, in that it necessarily depends on regional trade partners running trade deficits [80].

Finally, structural adjustment affects *broader psychosocial dynamics*. For instance, changes to social and labour policies can heighten psychosocial stress, with implications on health outcomes, including child and maternal health; or, alternately, prompt greater social cohesion as communities work to overcome adversity [34]. Adjustment policy may also provoke social unrest, thereby exacerbating existing social, economic and health problems [7].

## Conclusions

This article systematically reviewed empirical literature on the aggregate effect of structural adjustment programmes administered by the IMF, World Bank, and AfDB on child and maternal health in the developing world. The findings were contextualised with a discussion of the specific mechanisms involved. A detrimental association between structural adjustment policies and child and maternal health outcomes was found in 11 of 13 empirical studies reviewed; however, academic knowledge on which policies are producing or counteracting the aggregate effect is limited. It is also important to note that the overall detrimental effect of structural adjustment does not eliminate the possibility for beneficial pathways; rather, beneficial effects are outweighed at present by detrimental effects. Nevertheless, the almost unanimous identification of a detrimental effect among existing studies ought to compel IFIs to acknowledge and address health and social indicators in a much more systematic manner than previous adjustment packages have done.

This study is subject to a number of limitations. First, poor data collection in the developing world constrained many of the studies reviewed. While we have made this explicit in all relevant cases, the volume of studies containing non-trivial methodological flaws is such that evidence should be considered provisional. In particular, 6 of the 13 empirical studies do not adequately account for the non-random selection of countries into IMF programmes, which could bias findings. This choice in methodology is primarily driven by constraints to the study design due to lack of time-series data, as the health outcome are typically only reported on a single year, at two periods, or on a 5-yearly basis. Second, empirical studies specifically linking structural adjustment to child and maternal health outcomes are few, and are authored by a small number of scholars. Future research by independent research teams can increase confidence in findings. Third, empirical studies so far do not give adequate attention to evaluating each conceptual pathway, constraining the ability to advise on precisely *how* these programmes should be remodelled. Fourth, data availability is such that only one empirical study addressed a non-mortality outcome. Our findings are therefore not representative of alternative morbidities. Finally, this study is not exempt from the possibility of meta-biases, such as publication bias toward statistically significant effects, and positive reporting bias by IFI-affiliated authors.

IFIs have an obligation to ensure that universally agreed goals—like the SDGs—are an integral part of all policy efforts. Our review suggests that, in their current form, structural adjustment programmes are incongruous with achieving SDGs 3.1 and 3.2, which stipulate reductions in neonatal, under-5, and maternal mortality rates. It is telling that even the IMF’s Independent Evaluation Office, in assessing the performance of structural adjustment loans, noted that “outcomes such as maternal and infant mortality rates have generally not improved” [81]. From a public health perspective, this admission—in tandem with existing evidence—warrants a fundamental rethinking to the ways in which adjustment loans operate. Social goals are currently side-lined to fiscal targets, while detrimental effects are insufficiently acknowledged [22-27; 84]. The mechanisms identified in this review should serve as a guide for recalibrating structural adjustment programmes to protect children and mothers. In particular, future adjustment packages should be designed with population health as a core consideration. This entails a shift from *managing* negative social effects caused by adjustment policies—for instance, via poorly enforced social and priority spending targets— to *avoiding* policies that pose risks to social outcomes altogether [22]. IFIs must also conform to current objectives of the international community vis-à-vis health policy in support of universal health coverage, rather than continuing to endorse targeted social assistance [82].

We note several ways forward from this review. First, it is critical for studies assessing structural adjustment programmes to delineate programme effects from selection effects. Current literature is limited by the relatively narrow pool of studies that meet this criterion. Second, studies can improve policy relevance by producing more nuanced measurements for structural adjustment than the dummy variable approach currently deployed to indicate the mere presence of a programme. For instance, recent datasets now enable scholars to distinguish the effects of different conditionality policy mixes, in recognition of the relative heterogeneity of structural adjustment programmes [10, 22]. Third, and relatedly, future research is needed that examines the effects of specific policy mechanisms in structural adjustment programmes on child and maternal health outcomes; and these outcomes should extend beyond mortality measures to capture morbidities of the living. Finally, while cross-country study designs are a useful model of analysis, individual-level surveys may constitute a rich new area of exploration.

### Additional file

Additional file 1: PRISMA checklist. (DOC 56 kb)

## Appendix

**Table 3:** PubMed/Medline search strategy

i. Intervention terms
(“IMF”[Title/Abstract] OR “International Monetary Fund”[Title/Abstract] OR “World Bank”[Title/Abstract] OR “structural adjustment”[Title/Abstract] OR “structural adjustments”[Title/Abstract] OR “adjustment”[Title/Abstract] OR “austerity”[Title/Abstract] OR “African Development Bank”[Title/Abstract] OR “AfDB”[Title/Abstract] OR “neoliberal*”[Title/Abstract] OR “austerity”[Title/Abstract] OR “privatization”[MeSH Terms] OR “currency devaluation”[Title/Abstract] OR “Washington Consensus”[Title/Abstract] OR “International Financial Institution”[Title/Abstract] OR “International Organisation”[Title/Abstract])
ii. Outcome terms
AND ("fetal mortality"[MeSH Terms] OR "infant mortality"[MeSH Terms] OR "child mortality"[MeSH Terms] OR "maternal mortality"[MeSH Terms] OR "perinatal mortality"[MeSH Terms] OR "perinatal death"[MeSH Terms] OR "infant welfare"[MeSH Terms] OR "child welfare"[MeSH Terms] OR "maternal welfare"[MeSH Terms] OR "pregnancy complications"[MeSH Terms] OR "fetal nutrition disorders"[MeSH Terms] OR "infant nutrition disorders"[MeSH Terms] OR "child nutrition disorders"[MeSH Terms] OR “immunization”[MeSH Terms] OR “Infant Health”[MeSH Terms] OR “Child Health”[MeSH Terms] OR “Maternal Health”[MeSH Terms] OR “disease”[MeSH Terms] OR "Infant, Low Birth Weight"[Mesh Terms] OR “Infant, Postmature”[MeSH Terms] OR “Infant, Premature”[MeSH Terms] OR “HIV”[MeSH Terms] OR “Absenteeism”[MeSH Terms] OR “insurance coverage”[MeSH Terms])
iii. Setting terms
AND ("developing countries"[MeSH Terms] OR "africa south of the sahara"[MeSH Terms] OR "asia"[MeSH Terms] OR "latin america"[MeSH Terms] OR "europe, eastern"[MeSH Terms]) AND (("2000/01/01"[PDAT] : "3000/12/31"[PDAT]) AND "humans"[MeSH Terms] AND English[lang])

## Abbreviations

AfDB: African Development Bank
IFI: International financial institution
IMF: International Monetary Fund
MDG: Millennium Development Goals
NGO: Non-governmental organisation
SDG: Sustainable Development Goals
